# The Metropolitan Hospital Sunday Fund, 1890

**Published:** 1890-08-02

**Authors:** 


### The Metopolitan Hospital Sunday Fund, 1890.

### AWARDS AND CRITICISM.

The annual general report of the Committee of Distribution, which was adopted by the Council of the Metropolitan Hospital Sunday Fund on Monday, July 28th, is as follows :—

"Your committee have the honour to recommend the payment of awards to the 169 institutions enumerated in the appendix to this report, being eight more than last year, and an increase of 64 since the first awards were made in 1873.

"The total amount available for distribution, after allowing sufficient for liabilities and the usual current expenses, is £41,061 9s. 2d. Of this total £39,101 9s. 2d. is now recommended to 113 hospitals and 56 dispensaries.

"Five per cent. of the total collected—viz., £2,050—is set apart to purchase surgical appliances, in obedience to Law XII., in monthly proportions during the ensuing year.

"Your committee recommend that all payments to the fund after this date be carried to the credit of next year's Fund.

"In compliance with an order of the council, and for the special use of its members, tables of statistics have been prepared as usual, showing an analysis of the number of beds in hospitals, the cost of patients both at hospitals and dispensaries, the proportionate expense of management, as well as other valuable information, and copies are now ordered to be sent to every member of the council.

"The number of deputations representing committees of various hospitals, &c., invited to confer with your committee, and to offer explanations on matters of apparently unsatisfactory character, was sixteen. Of these, seven elected to leave their awards entirely in the hands of this committee. Nine were invited to attend a conference, three did not attend, and consequently are recommended for awards on reduced basis. From one hospital the committee received a satisfactory written explanation. Four hospitals sent deputations, and in three cases the committee raised the proposed basis. A deputation from one dispensary was also received. Your committee regret, however, that they have not felt justified in the case of one hospital to recommend it any award. Another hospital, whose basis worked out to a single unit, is not, therefore, recommended for an award.

"Your committee have also received applications from the Bath Mineral Water Hospital, which is considered to be too remote from London; and from other institutions which, being neither hospitals nor dispensaries, are ineligible. One dispensary and two convalescent hospitals sent in their statistics too late to be considered.

"Mansion House, 18th July, 1890."

### PARTICULARS OF THE AWARDS FOR 1890.

### Twenty-two General Hospitals.

Charing Cross, West Strand, W.C., £1,010 8s. 4d.; French, Lisle Street, W.C., £197 18s. 4d.; German, Dalston Lane, E., £677 1s. 8d.; Great Northern Central, Caledonian Road, N., £312 10s.; Guy's, £312 10s.; Italian, Queen Square, W.C., £93 15s.; King's College, Lincoln's Inn Fields, W.C., £1,406 5s.; London, Whitechapel, E., £3,125; Metropolitan, Kingsland Road, N., £312 10s.; Miller Hospital and Royal Kent Dispensary, £281 5s.; North-West London, Kentish Town Road, N.W., £312 10s.; Poplar, East India Road. E., £218 15s.; Royal Free, Gray's Inn Road, W.C., £947 18s. 4d.; St. George's, Hyde Park Corner, S.W., £1,562 10s.; SS. John and Elizabeth, Great Ormond Street, W.C., £125; St. Mary's, Paddington, W., £2,083 6s. 8d.; Seamen's, Greenwich, S.E., £708 6s. 8d.; The Middlesex, Charles Street, W., £2,083 6s. 8d.; Tottenham Training Hospital, Tottenham, N., £302 1s. 8d.; University College, Gower Street, W.C., £1,250; West London, Hammersmith, W., £572 18s. 4d.; Westminster, Broad Sanctuary, S.W., £1,145 16s. 8d.

### Special Hospitals.

Five Chest Hospitals.—City of London Hospital for Diseases of the Chest, Victoria Park, E., £937 10s.; Hospital for Consumption, Brompton, S.W., £1,562 10s.; North London Consumption Hospital, Hampstead, £364 11s. 8d.; Royal Hospital for Diseases of the Chest, City Road, £395 16s. 8d.; Royal National Hospital for Consumption (Ventnor), £260 8s. 4d.

Twelve Children's Hospitals.—Alexandra, Queen Square, W.C., £208 6s. 8d.; Belgrave, 1, Cumberland Street, Pimlico, S.W., £145 16s. 8d.; Cheyne Hospital for Children, Chelsea, S.W., £114 11s. 8d.; East London, Shadwell, E., £416 13s. 4d.; Evelina, Southwark Bridge Road, S.E., £489 11s. 8d.; Home for Children, Maida Vale, N.W., £88 10s. 10d.; Home for Sick Children and South London Dispensary for Women, Sydenham, S.E., £145 16s. 8d.; Hospital for Sick Children, Great Ormond Street, W.C., £781 5s.; North Eastern, 327, Hackney Road, E., £343 15s.; Paddington Green Hospital for Sick Children, £161 9s. 2d.; Victoria, Queen's Road, Chelsea, S.W., £354 3s. 4d.; "The Vine," Sevenoaks, £36 9s. 2d.

Four Lying-in Hospitals.—British, Endell Street, W.C., £52 1s. 8d.; City of London, City Road, E.C., £52 1s. 8d.; General, York Road, Lambeth, S.E., £78 2s. 6d.; Queen Charlotte's, Marylebone Road, W., £312 10s.

Six Hospitals for Women.—Chelsea Hospital for Women, Fulham Road, S.W., £244 15s. 10d.; Hospital for Women, Soho Square, W., £364 11s. 8d.; Grosvenor Hospital for Women and Children, Vincent Square, S.W., £72 18s. 4d.; New Hospital for Women, 322, Marylebone Road, W., £135 8s. 4d.; Royal Hospital for Diseases of Children and Women, Waterloo Bridge Road, S.E., £229 3s. 4d.; Samaritan Free, Lower Seymour Street, W., £489 11s. 8d.

Twenty-six other Special Hospitals.—Cancer, Brompton, S.W., £520 16s. 8d.; London Fever, Liverpool Road, N., £468 15s.; Gordon Hospital for Fistula, Vauxhall Bridge Road, £31 5s.; St. Mark's (Fistula), City Road, E.C., £104 3s. 4d.; National, for Diseases of the Heart, Soho Square, W., £135 8s. 4d.; Female Lock, Westbourne Green, W., £270 16s. 8d.; Male Lock, Dean Street, Soho, W., £57 5s. 10d.; Hospital for Epilepsy, Paralysis, and other Diseases of the Nervous System, Portland Terrace, N.W., £104 3s. 4d.; National, for the Paralyzed and Epileptic, Queen Square, W.C., £614 11s. 8d.; West End Hospital, Welbeck Street, W., *nil*; Central London Ophthalmic, Gray's Inn Road, W.C., £62 10s.; Royal London Ophthalmic, Moorfields, E.C., £572 18s. 4d.; Royal South London Ophthalmic, St. George's Circus, S.E., £88 10s. 10d.; Royal Westminster Ophthalmic, King William Street, W.C., £116 13s. 4d.; Western Ophthalmic, Marylebone, W., £22 18s. 4d.; City Orthopædic, Hatton Garden, E.C., £72 18s. 4d.; National Orthopædic, Great Portland Street, W., £41 13s. 4d.; Royal Orthopædic, Hanover Square, W., £93 15s.; Hospital for Diseases of the Skin, Stamford Street, S.E., £20 16s. 8d.; Central London Throat and Ear, Gray's Inn Road, W.C., £57 5s. 10d.; Royal Ear Hospital, Frith Street, Soho, W., £4 3s. 4d.; Hospital for Diseases of the Throat, Golden Square, W., *nil*; London Homœopathic, Great Ormond Street, W.C., £234 7s. 6d.; London Temperance, Hampstead Road, N.W., £468 15s.; The Dental, Leicester Square, W.C., £98 19s. 2d.; National Dental, 149, Great Portland Street, W., £10 8s. 4d.

### Twenty Convalescent Hospitals.

Metropolitan Convalescent Institution, Walton-on-Thames, &c., £677 1s. 8d.; Bexhill-on-Sea Convalescent Home, £302 1s. 8d.; All Saints' Convalescent Home, Eastbourne, £552 1s. 8d.; Mrs. Gladstone's Convalescent Home, Woodford, E., £156 5s.; Hanwell Convalescent Home, Middlesex, £20 16s. 8d.; Herbert Convalescent Hospital, Bournemouth, £36 9s. 2d.; King's College Convalescent Hospital, Hemel Hempstead, £134 2s.; Mrs. Kitto's Convalescent Hospital, Reigate, Surrey, £83 6s. 8d.; Mrs. Marshman's Convalescent Hospital, Brighton, £78 2s. 6d.; Mary Wardell Convalescent Home, Great Stanmore, £145 16s. 8d.; Morley Convalescent Hospital, St. Margaret's, Kent, £62 10s.; Princess Frederica's Convalescent Home, East Molesey, £31 5s.; Ramsgate Children's (Boys only) Convalescent Home, £20 16s. 8d.; St. Andrew's Convalescent Home, Clewer, £166 13s. 4d.; St. Andrew's Convalescent Home, Folkestone, £166 13s. 4d.; St. Barnabas Convalescent Home, Ramsgate, £10 8s. 4d.; St. Leonards-on-Sea Convalescent Hospital for Children, £104 3s. 4d.; St. Mary Magdalen Convalescent Home, Paddington, W., £52 1s. 8d.; St. Michael's Convalescent Home, Westgate-on-Sea, £41 13s. 4d.; Seaside Convalescent Hospital, Seaford, £156 5s.

### Eleven Cottage Hospitals.

Beckenham, £52 1s. 8d.; Blackheath and Charlton, £67 14s. 2d.; Burstead, £20 16s. 8d.; Chislehurst, &c., £52 1s. 8d.; Eltham, £31 5s.; Enfield, £31 5s.; Epsom and Ewell, £46 17s. 6d.; Reigate and Redhill, £72 18s. 4d.; Shedfield, £8 6s. 8d.; Sidcup, £26 0s. 10d.; Wimbledon, £36 9s. 2d.

### Seven Institutions.

Establishment for Gentlewomen, Harley Street, W., £83 6s. 8d.; Hampstead Home Hospital, £62 10s.; National Sanatorium, Bournemouth, £72 18s. 4d.; Royal Sea Bathing Infirmary, Margate, £604 3s. 4d.; Invalid Asylum, Stoke Newington. £78 2s. 6d.; "Firs Home," Bournemouth, £31 5s.; "St. Catherine's Home," Ventnor, £52 1s. 8d.

### Fifty-Six Dispensaries.

Battersea (Provident), £57 5s. 10d.; Brixton, &c., £46 17s. 6d.; Brompton (Provident), £26 0s. 10d.; Camberwell (Provident), £93 19s. 2d.; Camden Town (Provident), £10 8s. 4d.; Chelsea, £46 17s. 6d.; Child's Hill (Provident), £10 8s. 4d.; City, £119 15s. 10d.; City of London and East London, £26 0s. 10d.; Clapham (General), £36 9s. 2d.; Deptford (Provident), £12 10s.; Eastern, £31 5s.; Farringdon, £46 17s. 6d.; Finsbury, £57 5s. 10d.; Forest Hill, £36 9s. 2d.; Gipsy Hill (Provident), £20 16s. 8d.; Hackney (Provident), £6 5s.; Hampstead (Provident), £52 1s. 8d.; Holloway and North Islington, £72 18s. 4d.: Islington, £57 5s. 10d.; Kensal Town (Provident), £13 10s. 10d.; Kensington, £83 6s. 8d.; Kilburn, &c., £48 19s. 2d.; Kilburn (Provident), £31 5s.; London Street, Spitalfields, E., £20 16s. 8d.; London Medical Mission, Endell Street, W.C., £52 1s. 8d.; Margaret Street Infirmary for Consumption, W., £10 8s. 4d.; Metropolitan (Fore Street, E.C.), £41 13s. 4d.; Notting Hill (Provident), £26 0s. 10d.; Paddington (Provident), £41 13s. 4d.; Pimlico (Provident), Lupus Street, S.W., £15 12s. 6d.; Portland Town (Free), £20 16s. 8d.; Portobello Road (Provident), £5 4s. 2d.; Public, Stanhope Street, W.C., £57 5s. 10d.; Queen Adelaide's, Bethnal Green, E., £41 13s. 4d.; Royal General, Bartholomew Close, E.C., £52 1s. 8d.; Royal Maternity Charity, Finsbury Square, E.C., £104 3s. 4d.; Royal Pimlico (Provident), £57 5s. 10d.; Royal South London, St. George's Circus, S.E., £62 10s.; SS. George's and James's, King Street, W., £62 10s.; St. George's, Hanover Square (Provident), £52 1s. 8d.; St. John's Wood (Provident), £22 18s. 4d.; St. Marylebone, General, £36 9s. 2d.; St. Pancras and Northern, £41 13s. 4d.: SS. Paul and Barnabas (Provident), Pimlico, S.W., £52 1s. 8d.; South Lambeth, £62 10s.; South London Medical Aid Institute, Waterloo Bridge Road, S.E., £26 0s. 10d.; Stamford Hill, &c., Stoke Newington, N., £52 1s. 8d.: Tower Hamlets, Stepney, E., £52 1s. 8d.; Wandsworth Common (Provident), £10 8s. 4d.; Westbourne (Provident), £20 16s. 8d.; West Dulwich (Provident), £5 4s. 2d.; Western, Rochester Row, Westminster, S.W., £41 13s. 4d.; Western General, Marylebone Road, S.W., £135 8s. 4d.; West Ham and Stratford, E., £62 10s.; and Westminster General, Gerrard Street, W., £36 9s. 2d.

